# Mathematical Modelling of COVID-19 Transmission in Kenya: A Model with Reinfection Transmission Mechanism

**DOI:** 10.1155/2021/5384481

**Published:** 2021-10-16

**Authors:** Isaac Mwangi Wangari, Stanley Sewe, George Kimathi, Mary Wainaina, Virginia Kitetu, Winnie Kaluki

**Affiliations:** ^1^Bomet University College, School of Pure and Applied Sciences, Department of Mathematics and Computer Science, P.O. Box 701 20400, Bomet, Kenya; ^2^The Catholic University of Eastern Africa (CUEA), Department of Mathematics and Actuarial Science, I Langata Main Campus I Bogani East Rd, Off Magadi Rd, P.O. Box 62157-00200 Nairobi, Kenya

## Abstract

In this study we propose a Coronavirus Disease 2019 (COVID-19) mathematical model that stratifies infectious subpopulations into: infectious asymptomatic individuals, symptomatic infectious individuals who manifest mild symptoms and symptomatic individuals with severe symptoms. In light of the recent revelation that reinfection by COVID-19 is possible, the proposed model attempt to investigate how reinfection with COVID-19 will alter the future dynamics of the recent unfolding pandemic. Fitting the mathematical model on the Kenya COVID-19 dataset, model parameter values were obtained and used to conduct numerical simulations. Numerical results suggest that reinfection of recovered individuals who have lost their protective immunity will create a large pool of asymptomatic infectious individuals which will ultimately increase symptomatic individuals with mild symptoms and symptomatic individuals with severe symptoms (critically ill) needing urgent medical attention. The model suggests that reinfection with COVID-19 will lead to an increase in cumulative reported deaths. Comparison of the impact of non pharmaceutical interventions on curbing COVID19 proliferation suggests that wearing face masks profoundly reduce COVID-19 prevalence than maintaining social/physical distance. Further, numerical findings reveal that increasing detection rate of asymptomatic cases via contact tracing, testing and isolating them can drastically reduce COVID-19 surge, in particular individuals who are critically ill and require admission into intensive care.

## 1. Introduction

The world is currently in the midst of a COVID-19 pandemic. In December 2019 the Wuhan city of China was considered as the epicenter of the novel coronavirus. The pandemic unfolded as a cluster of patients being admitted to hospital in late December 2019. These patients were diagnosed with pneumonia [1]. At first the medical practitioners linked the cause of the disease to a seafood and wet animal market in Wuhan, Hubei Province, China [1]. As it is now known the aetiological agent of the disease is a novel coronavirus identified as Severe Acute Respiratory Syndrome Coronavirus 2 (SARS-CoV-2) and the disease caused by the virus was named by the World Health Organization (WHO) as Coronavirus Disease 2019 (COVID-19) [2]. WHO declared COVID-19 a pandemic in March 2020 [2]. As at Mid July 2020 COVID-19 had spread to over 213 countries causing about 15,969,465 infections and 643,390 deaths.

Similar to two other coronaviruses that in recent years triggered major outbreaks in humans (namely, the Severe Acute respiratory Syndrome Coronavirus 1 (SARS-CoV-1) and the Middle Eastern Respiratory Syndrome Coronavirus (MERS-CoV) [3, 4]), COVID-19 is transmissible from human-to-human through direct contact with objects or surfaces that are contaminated with the virus. Moreover, inhalation of respiratory droplets from both asymptomatic and symptomatic infectious individuals causes transmission [5]. The scientific community via WHO reported that the virus can also be exhaled through normal breathing and this ultimately leads to new infections. COVID-19 has an incubation period ranging from 2-14 days with approximately 97.5% of infected people manifesting disease symptoms within 11.5 days of infection [6–9]. Generally a larger proportion of infected individuals exhibit mild symptoms or no symptoms [2].

The global spread and unstoppable nature of COVID-19 compelled China and many nations to institute draconian containment measures [10]. As the pandemic unfolds there are currently several vaccines in use, including AstraZeneca, Johnson and Johnson, Modena, Pfizer etc, approved by the WHO for the management of COVID-19. However, for many developing countries, getting the vaccines to administer to significant proportions of their populations has proven to be a tall order due to high demand among developed countries where these vaccines are manufactured. Consequently, in these developing countries efforts aimed at mitigating COVID-19 are mainly focusing on non-pharmaceutical interventions which include; using face masks, social-distancing, quarantine of suspected cases and contact tracing [11]. These non-pharmaceutical containment measures proved to be a success in some countries, e.g., in Wuhan City in China, while the same measures failed in some countries probably due to non adherence to the measures by the general populace, individual irresponsibility or inefficient contact tracing of asymptomatic and symptomatic cases [11]. On the other hand, in developed countries where vaccines are available more than 60% of their population have received the first dose of the vaccine [12].

In Kenya, the first case (index case) of COVID-19 positive was reported on 13 March, 2020, with Nairobi being the epicenter. In the following months of April and May the COVID-19 increased at a slow pace creating an illusion that the containment measures that were put in place by the Kenyan Government were a success. This resulted to even the government setting the date of reopening of schools and partial lifting of strict measures on June 1, 2020. However, this never happened as COVID-19 started to spread rapidly at the end of May, prompting the Government to rethink its containment strategies. As of 27 July, 2020, 43 counties in Kenya had COVID-19 cases with about 17,975 infections, 7,833 discharged cases (recoveries) and 290 deaths. During that same time, Nairobi county (capital city) had the highest COVID-19 cases (about 12,500). As on June 7, 2021, the total number of positive cases reported in Kenya was 172,325 while the total number of fatalities reported was 3,264. The Kenyan government has continued to urge its citizens to adhere to the various non-pharmaceutical interventions as it awaits for more vaccines. Meanwhile countrywide community transmission ccontinuesto unfold.

Both modellers and medical practitioners acknowledge that COVID-19 pandemic is compounded with a multitude of challenges. First the epidemiological characteristics of the COVID-19 is currently unfolding and this is yet to be entirely elucidated [1]. The available scientific evidence classify COVID-19 infected individuals into three broad cohorts: individuals who manifest severe symptoms, individuals who manifest mild symptoms and those individuals who do not manifest any COVID-19 symptoms (asymptomatic) and yet they remain infectious. The non-manifestation of COVID-19 symptoms amongst some infected people complicates the epidemiology of the COVID-19 pandemic. First asymptomatic individuals are unlikely to seek medical care or self-quarantine given that they cannot tell whether they have the disease unless confirmed through testing or contact tracing. Secondly, they will continue interacting with healthy people thereby spreading the virus. Again although the asymptomatic cohort form the large proportion of COVID-19 infections, it is not yet known to what extent they spread the virus relative to cohorts with severe symptoms which constitute a small proportion of COVID-19 infections. Questions that are currently asked by many medical professionals regarding asymptomatic cases include:

- Could the asymptomatic cases be the one that will lead to a prolonged COVID-19 pandemic?
- Could the asymptomatic cases lead to sporadic outbreaks in future?

According to the findings published by [13], more than half of new infections of COVID-19 are attributed to people not exhibiting symptoms (pre-symptomatic and asymptomatic). In their work they suggested that approximately 40% of people infected with COVID-19 are asymptomatic. The fact that people can transmit the virus without knowing complicates epidemiological dynamics of COVID-19.

As the pandemic continues to spread there remains many unsolved questions. The initial assumption that recovered patients developed a long lasting protective immunity lead to disagreement among distinguished scientists, (see e.g., [14]). This is due to the fact that the duration of protective immunity will not only impact the epidemiological dynamics of the current pandemic but also the post-pandemic period. At some point some researchers suggested that recovered individuals need to be classified as so-called, “immunity passport” and be allowed to relax social distancing measures. According to [15], it was argued that such an action would provide data on levels of herd immunity in the population. The recent emergence of SARS-CoV-2 and the limited scale at which SARS-CoV-1 and MERS-CoV epidemics occurred obscure the availability of data which could act as a concrete evidence of reinfection by SARS-CoV-2. However, reinfection with SARS-CoV-2 cannot be ruled out [14].

According to the recent published research on serological testing for seasonal Human Coronavirus229E (HCoV-229E) which investigated antibody dynamics after infection, they found that majority of patients lost 50% of their Nct-antibodies after a duration of six months, 75% after a year and completely returned to baseline 4 years post-infection [14]. Thus, from these findings, the prospect of gaining a functional herd immunity seems unreasonable. Furthermore, the rapid decline of protective immunity challenges the idea of herd immunity. Thus, once the short-lived immunity is lost; people can be reinfected once they are exposed to the same coronavirus or a new variant, (e.g., Delta variant). One of the research question conducted in this study stems from asking how reinfection with SARS-CoV-2 will impact COVID-19 dynamics in the long-term. Consequently, the challenge posed by the asymptomatic cases in conjunction with reinfection calls for further research that needs to forecast the likely disease burden, morbidity and mortality. We identify this as a gap that should be addressed urgently so as to project and predict COVID-19 dynamics in real time and also fully elucidate on the epidemiological characteristics of the COVID-19 pandemic.

Epidemiological models are increasingly becoming an important tool in helping understand the intricate dynamics governing the spread of infectious diseases [16–20]. Numerous mathematical models attempting to unravel the population dynamics of COVID-19 have been formulated and this trend is ongoing, for instance see [21–25]. In the current study, we propose a mathematical model for COVID-19 in human population with the aim of examining the following:

- How does silent transmission (asymptomatic transmission) relative to the symptomaticcase transmission impact the long term epidemiological dynamics of COVID-19?
- In light of the recent revelation that reinfection by COVID-19 is possible, how willreinfection alter the future dynamics of the recent unfolding pandemic?

The mathematical model is fitted using Kenya COVID-19 data to project and predict the cumulative number of reported cases as well as give insights on the likely peak time for COVID-19 in Kenya in presence of reinfection infection mechanism.

## 2. Materials and Method

### 2.1. Construction of COVID-19 Mathematical Model

In the spirit of Kermack-McKendrick-type mathematical models [16, 26, 27] that track the transmission dynamics of infectious diseases, we constructed and analyzed a COVID-19 model in a human population (see also [21, 24]). The total human population at time *t* denoted by P_h_ is stratified into nine mutually exclusive subpopulations, namely; the susceptible (*S*(*t*)), the exposed subpopulation, (*E*(*t*)), asymptomatically infectious individuals (*I_a_*(*t*)), symptomatically infectious individuals with mild symptoms (*I_m_*(*t*)), symptomatically infectious individuals who manifest severe symptoms *I_s_*(*t*), hospitalized individuals (*H*(*t*)), detected infectious individuals through contact tracing and mass testing *I_d_*(*t*) and recovered individuals (*R_I_t*) and *R_L_*(*t*)*. R_I_*(*t*) represents recently recovered individuals with high levels of COVID-19 antibodies. However, the protective immunity acquired is not permanent. With time these individuals lose the protective immunity and progress to *R_L_*(*t*) class which represent recovered individuals with weak or zero immunity. Thus, the total human population is given as

(1) $$\begin{array}{c} {\text{P}}_{\text{h}}\left(\text{t}\right)=\text{S}\left(\text{t}\right)+\text{E}\left(\text{t}\right)+{\text{I}}_{\text{a}}\left(\text{t}\right)+{\text{I}}_{\text{m}}\left(\text{t}\right)+{\text{I}}_{\text{s}}\left(\text{t}\right)+\text{H}\left(\text{t}\right)+{\text{I}}_{\text{d}}\left(\text{t}\right)+{\text{R}}_{\text{I}}\left(\text{t}\right)+{\text{R}}_{\text{L}}\left(\text{t}\right). \end{array}$$

The mathematical model governing the transmission dynamics of COVID-19 in humans is described by a system of deterministic nonlinear differential equations given in equation (2).

(2) $$\begin{array}{c} \begin{array}{c} \frac{dS}{dt}=-\lambda S,\\ \frac{dE}{dt}=\lambda S+\theta \lambda R_{L}-\sigma E,\\ \frac{{dI}_{a}}{dt}=f\sigma E-\left(\alpha +\xi +d_{a}+\gamma_{1}\right)I_{a},\\ \frac{{dI}_{m}}{dt}=\left(1-f\right)\sigma E+\xi I_{a}-\left(v+\eta_{1}+d_{m}+\gamma_{2}\right)I_{m},\\ \frac{{dI}_{s}}{dt}={vI}_{m}-\left(d_{s}+\eta_{2}\right)I_{s},\\ \frac{dH}{dt}=\eta_{1}I_{m}+\eta_{2}I_{s}-\left(\gamma_{3}+d_{h}\right)H,\\ \frac{{dI}_{d}}{dt}=\alpha I_{a}-\left(d_{c}+\gamma_{4}\right)I_{d},\\ \frac{{dR}_{I}}{dt}=\gamma_{1}I_{a}+\gamma_{2}I_{m}+\gamma_{3}H+\gamma_{4}I_{d}-\rho R_{I},\\ \frac{{dR}_{L}}{dt}=\rho R_{1}-\theta \lambda R_{L}, \end{array} \end{array}$$

where *λ* is the *force of infection* and is given by

(3) $$\begin{array}{c} \lambda =\frac{\left(1-\omega \right)\left(1-\kappa \right)\beta \left(I_{s}+\theta_{m}I_{m}+\theta_{a}I_{a}+\theta_{h}H\right)}{P_{h}-q\left(H+I_{d}\right)}. \end{array}$$

In equation (3) *β* represents the contacts that are capable of resulting to COVID-19 transmission (i.e., effective contact rate), *ω* is the fraction of the members of the public who correctly and consistently wear face masks in public. The parameter *ω* which is assumed to lie within 0 < *ω* ≤ 1, is the efficacy of the face masks in preventing community transmission of COVID-19. Values of *ω* close or equal to zero indicate that face masks are not effective in preventing widespread transmission of COVID-19 if worn by susceptible humans or in preventing transmission if worn by; infectious symptomatic, infectious asymptomatic with mild symptoms or infectious symptomatic with severe symptoms. On the other hand *ω* values close or equal to unity imply that use of face masks significantly reduces transmission or acquisition of COVID-19 in the community. Further, we introduced a parameter that account for reduction of COVID-19 transmission if individuals adhere to social-distancing (or physical distancing). According to WHO and MOH (Ministry of Health) COVID-19 protocols, individuals are required to maintain a minimum distance of at least one metre apart to prevent transmission of COVID-19. Hence, parameter *κ,* (with 0 < *κ* ≤ 1) represent the proportion of the population who strictly observe the minimum social distance required to prevent one from acquiring or transmitting COVID-19. The modification parameters 0 < *θ*_*m*_, *θ*_*a*_, *θ*_*h*_ ≤ 1, respectively, account for the relative infectiousness of symptomatic infectious individuals with mild symptoms *I_m_*, infectious asymptomatic individuals *Ia* and infectious hospitalized individuals *H* in relation to the infectious symptomatic individuals with severe symptomatics *I_s_* (i.e individuals manifesting severe symptoms and need hospitalization). The parameter *q* (0*<q*≤1) is a measure of how effective hospitalization of infectious individuals with severe symptoms and detection of asymptomatic individuals (via contact tracing and mass testing) reduce transmission of the COVID-19.

Upon coming into contact with an infectious COVID-19 individual, a person progresses to the exposed compartment *E,* where they sojourn for a certain period (i.e., intrinsic incubation period). A fraction of these individuals develop mild symptoms at a rate (1−*f*)*σ* while the rest transition to infectious asymptomatic individuals class at a rate *fσ.* Thus, 1*/σ* is the intrinsic incubation period for exposed individuals. The parameter *ξ* represents the rate at which infectious asymptomatic individuals develop mild symptoms and move to symptomatic-mild infectious class. The symptomatic-mild infectious humans manifest severe symptoms and progress to the class of infectious individuals with severe symptoms at a rate *v.* Infectious humans with mild symptoms are detected via contact tracing and mass testing and hospitalized at a rate *η*_1_ while infectious humans with severe symptoms are detected and hospitalized (move to class *H*) at a rate *η*_2_. The parameter *α* represents the rate at which infectious asymptomatic individuals are detected via contact tracing and isolated at home (home-based care) or quarantined such that they have no contact with the susceptible humans. The parameters *γi,* (*i*=1,2,3,4), respectively, represents the rates at which individuals residing in infectious asymptomatic (*I_a_*), infectious symptomatic-mild (*I_m_*), infectious hospitalized (*H*) and detected asymptomatic (*I_d_*) classes, recover and move to *R_I_* class (recovered individuals with temporary immunity). Moreover, humans in classes *I_a_,I_m_,I_s_,H,I_d_* experience COVID-19-induced mortality at rates *d_a_,d_m_,d_s_,d_h_,d_c_,* respectively. Individuals in *R_I_* class lose post-COVID-19 protective immunity at a rate *ρ* and move to class *R_L_* (which constitute humans with weak or no protective immunity). Hence, 1*/ρ* is the duration of protective immunity amongst recovered individuals, *R_I_.* Due to weak or no protective immunity individuals in *R_L_* become susceptible again and once exposed to infectious humans become reinfected at a rate *θλ.* The modification parameter 0*<θ*≤1 accounts for partial protection among humans in *R_L_* in relation to susceptible humans *S.* It is imperative to note that death due to COVID-19 is inevitable in Kenya, just like any other country experiencing the COVID-19 pandemic. Thus, we introduce the state variable *C_d_*(*t*) which measures the number of COVID-19 induced mortality in Kenya. This state variable is introduced for the purpose of calibrating our model with the readily available COVID-19 data, and also for quantifying and predicting the burden of COVID-19. Hence, from model equation (2), the rate at which COVID-19 infected individuals succumb to death is described by

(4) $$\begin{array}{c} \frac{{dC}_{d}}{dt}=d_{a}I_{a}+d_{m}I_{m}+d_{h}H+d_{d}I_{d}+d_{s}I_{s}. \end{array}$$

**Figure 1:**
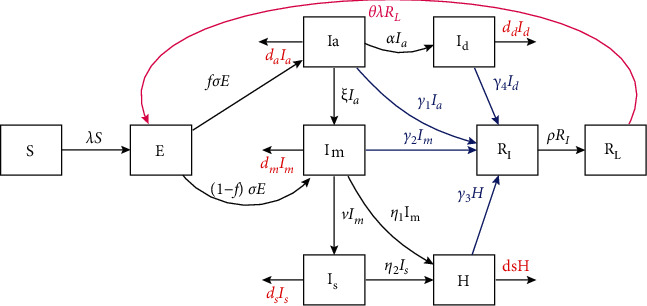
A schematic diagram of COVID-19 model equation (2). All arrows indicate transition from one disease status to another. Blue arrows show recovery rates for classes *I_a_,I_m_,I_d_,H* while the curved magenta arrow indicates reinfection pathway. Red arrows indicate COVID-19 induced mortality.

A schematic diagram for the COVID-19 mathematical model is shown in Figure 1. The state variables and parameters values are, respectively, tabulated in Tables 1 and 2.

The proposed model equation (2) is different from the recent model analyzed by [24] in that we split the symptomatic class into two cohorts depending on the severity of COVID-19 symptoms. That is we have incorporated into our model individuals with mild symptoms and individuals with severe symptoms. This is evidenced by COVID-19 documented literature, e.g., [10], which established that exposed individuals do not suddenly manifest severe symptoms rather progress at a slower pace from mild to severe symptoms to critically ill. According to our knowledge there is no other model that has attempted to understand the epidemiological impact of the duration of protective immunity among COVID-19 recoveries. Many COVID-19 models classify recovered individuals as one group with uniform and permanent protective immunity (see [10, 21, 25]). Perhaps the most distinguishable feature for the proposed COVID-19 model is the incorporation of reinfection pathway that so far has not been accounted for in any COVID-19 mathematical model despite evidence being available that reinfection can occur [14].

### 2.2. Baseline Values of Model Parameters

The baseline epidemiological parameter values of the proposed model are estimated from the readily available COVID-19 data and also from the existing published literature. The intrinsic incubation periods for COVID-19 have been estimated by some studies to range between 2 to 14 days, with approximately 97.5% manifesting clinical symptoms of COVID-19 within 11.5 days of infection [7–9]. From other documented sources the incubation period has been estimated to be about 5-6 days [6]. We thus consider an average incubation period of about 5.1 days which are taken from documented ranges. Hence, *σ*=1*/*5.1 per da*y* [7]. The rates at which asymptomatic infectious individuals manifest mild clinical symptoms of COVID-19 and thus progress to *I_m_* is taken to be *ξ* = 1/4*per* day [21]. Similarly, we shall set the rate at which infectious individuals with mild symptoms progress to severe symptomatic class *Is* to *v* = 1/4*per* day.

According to the studies conducted in [28, 29], viral shedding of COVID-19 amongst infected patients lasts about 10 days. Consequently, we set the recovery rates from COVID-19 infection to be *γ*_*i*_{*i* = 1, 2, 3, 4} = 1/10*per* day. The hospitalization rates of individuals manifesting mild and severe symptoms are estimated based on Ferguson et al. [28] study, who assumed that there is a short time lag of approximately 5 days between the onset of COVID-19 symptoms and the time humans seek medical attention (hospitalization). We thus set *η*_1_ = *η*_2_ = 1/5*per* day. The findings of some studies suggest that for most of COVID-19 infections, about 80% manifest mild or no symptoms [2, 29, 30] while about 14% manifest severe symptoms. Another proportion of about 6% show severe symptoms that require intensive care unit admissions. Here we assume that during early progression of COVID-19 infection 80% of all infections in Kenya are asymptomatic while the remaining have mild symptoms (thus, we set *f*=0.80 and 1−*f*=0.20). The modification parameter accounting for the relative infectiousness of infectious asymptomatic individuals was estimated to be 0.5 in [28, 31]. Moreover, Li et al. [32] estimated the same modification parameter to range between 0.42 and 0.55. Hence, we set *θ*_*m*_ = *θ*_*a*_ = *θ*_*h*_ = 0.48. The current mortality rate in Kenya is about 1.6% which is much lower in comparison to the world projections which indicates that about 10% of COVID19 patients die [33]. We estimate *d_h_*=*d_s_*=0.0016 *per* day and the mortality rates for those infectious asymptomatic individuals, infectious individuals with mild symptoms and detected asymptomatic class to be *d*_*a*_ = *d*_*m*_ = *d*_*d*_ = 0.5*d*_*h*_*per* day. The parameter *q* that accounts for the effectiveness of detection of asymptomatic individuals (via contact tracing) and hospitalization of individuals with mild and severe symptoms is estimated to be *q*=0.5. From the recent research on how long COVID-19 protective immunity last upon infection, it was found that majority of patients lost 50% of their Nct-antibodies after a duration of six months, 75% after a year and completely returned to baseline 4 years post-infection [14]. Thus we estimate *ρ*=1*/*180 *per* day. Based on the study of Ngonghala et al. [21] the rate at which infectious asymptomatic individuals are detected via contact tracing and isolated is set at *α*=0.1168. The remaining parameters (*β,κ,ω*) are estimated using the Kenya COVID-19 dataset.

## 3. Basic Properties of the Model

In this section, the dynamical properties of model equation (2) are qualitatively are analyzed.

### 3.1. Positivity and Boundedness

Since we are considering a human population the model equation (2) need to be epidemiolgically meaningful. Thus, we show that all state variables of model (2) are nonnegative for all time, *t >0* and that the region

(5) $$\begin{array}{c} \Omega =\left\{\left(S,E,I_{a},I_{m},I_{s},I_{d},H,R_{I},R_{L}\right)\in {\mathbb{R}}_{+}^{9}:S+E+I_{a}+I_{m}+I_{s}+I_{d}+H+R_{I}+R_{L}\leq P_{h}\left(0\right)\right\} \end{array}$$

is bounded. Hence, the following Theorem:

**Theorem 1 .**

Let the initial data supplied to the model (2) be; *S*(0) ≥ 0, *E*(0) ≥ 0, *I*_*a*_(0) ≥ 0, *I*_*m*_(0) ≥ 0, *I*_*s*_(0) ≥ 0, *I*_*d*_(0) ≥ 0, *H*(0) ≥ 0, *R*_*I*_(0) ≥ 0, *R*_*L*_(0) ≥ 0. Then the solution for model (2) remain positive for all time, *t* > 0, in the region *Ω.*

**Proof**

Let *t*_1_ = sup{*t* > 0 : *S* > 0, *E* > 0, *I*_*a*_ > 0, *I*_*m*_ > 0, *I*_*s*_ > 0, *I*_*d*_ > 0, *H* > 0, *R*_*I*_ > 0, *R*_*L*_ > 0 ∈ [0, *t*]}. Thus, *t*_1_ > 0. Considering the first equation of the model equation (2), we have

(6) $$\begin{array}{c} \frac{dS}{dt}=-\lambda S,\text{where} \end{array}$$

remains as defined in equation (3). Equation Equation (6) can be re-written as

(7) $$\begin{array}{c} \int \frac{dS}{dt}=\int \lambda S, \end{array}$$

so that

(8) $$\begin{array}{c} S\left(t_{1}\right)=S\left(0\right)\exp\left[-\int_{0}^{t_{1}}\lambda \left(\tau d\tau \right)\right]>0. \end{array}$$

Following a similar procedure we can verify that *E* > 0, *I*_*a*_ > 0, *I*_*m*_ > 0, *I*_*s*_ > 0, *I*_*d*_ > 0, *H* > 0, *R*_*I*_ > 0, *R*_*L*_ > 0. Thus, all the trajectories of model equation (2) remain positive for all nonnegative initial conditions.

**Lemma 1 .**

The region *Ω* = {(*S*, *E*, *I*_*a*_, *I*_*m*_, *I*_*s*_, *I*_*d*_, *H*, *R*_*I*_, *R*_*L*_) ∈ ℝ_+_^9^ : *S* + *E* + *I*_*a*_ + *I*_*m*_ + *I*_*s*_ + *I*_*d*_ + *H* + *R*_*L*_ ≤ *P*_*h*_(0)} is positively invariant and absorbing with respect to the set of nonlinear differential equations of model (2).

**Proof**

We show that the biologically meaningful solutions of model (2) are uniformly bounded in the region *Ω.* Let *S,E,I_a_,I_m_,I_d_,H,R_I_,R_L_* be solutions of model system (2) obtained upon supplying model (2) with nonnegative initial conditions. It is not difficult to note that the total population P_h_, fulfils the inequality

(9) $$\begin{array}{c} \frac{{dP}_{h}}{dt}=-\left(d_{a}I_{a}+d_{m}I_{m}+d_{s}I_{s}+d_{h}H+d_{d}I_{d}\right) \end{array}$$

Equation (9) can be re-written as

(10) $$\begin{array}{c} \frac{{dP}_{h}}{dt}\leq -\bar{\delta }P_{h}\;\text{where }\bar{\delta }=\min\left(d_{a},d_{m},d_{s},d_{h},d_{d}\right). \end{array}$$

Thus,

(11) $$\begin{array}{c} \frac{{dP}_{h}}{dt}\leq -\bar{\delta }P_{h}. \end{array}$$

which implies that

(12) $$\begin{array}{c} P_{h}\left(t\right)\leq P_{h}\left(0\right)e^{-\bar{\delta }t}. \end{array}$$

Note that P_h_(*t*) tends to P_h_(0) as *t*→∞. Hence, the region Ω attracts all solutions in ℝ_+_^9^.

### 3.2. Asymptotic Stability Analysis of the Disease-Free Equilibrium

The model (2) has a disease-free equilibrium *𝒟* = (*S*^∗^, *E*^∗^, *I*_*a*_^∗^, *I*_*m*_^∗^, *I*_*s*_^∗^, *I*_*d*_^∗^, *H*^∗^, *R*_*I*_^∗^, *R*_*L*_^∗^) = (*S*(0), 0, 0, 0, 0, 0, 0, 0, 0) wher *S*(0) represent the initial size of the population that is susceptible to COVID-19. The asymptotic stability of the disease free equilibrium will be analyzed using the next generation operator method [34, 35]. The next generation operator matrices, F and V representing new infection terms and the transition terms are, respectively, given by

(13) $$\begin{array}{c} F=\left(\begin{array}{cccccc} 0 & \left(1-\omega \right)\left(1-\kappa \right){\beta \theta }_{a} & \left(1-\omega \right)\left(1-\kappa \right){\beta \theta }_{m} & \left(1-\omega \right)\left(1-\kappa \right)\beta & \left(1-\omega \right)\left(1-\kappa \right){\beta \theta }_{h} & 0\\ 0 & 0 & 0 & 0 & 0 & 0\\ 0 & 0 & 0 & 0 & 0 & 0\\ 0 & 0 & 0 & 0 & 0 & 0\\ 0 & 0 & 0 & 0 & 0 & 0\\ 0 & 0 & 0 & 0 & 0 & 0 \end{array}\right)\\ V=\left(\begin{array}{cccccc} \sigma & 0 & 0 & 0 & 0 & 0\\ -f\sigma & K_{1} & 0 & 0 & 0 & 0\\ -\left(1-f\right)\sigma & -\xi & K_{2} & 0 & 0 & 0\\ 0 & 0 & -v & K_{3} & 0 & 0\\ 0 & 0 & -\eta_{1} & -\eta_{2} & K_{4} & 0\\ 0 & -\alpha & 0 & 0 & 0 & K_{5} \end{array}\right), \end{array}$$

where *K*1 = (*α* + *ξ* + *d*_*a*_ + *γ*_1_), *K*_2 = (*v* + *η*_1_ + *d*_*m*_ + *γ*_2_), *K*_3 = (*d*_*s*_ + *η*_2_), *K*_4 = (*d*_*h*_ + *γ*_3_), *K*_5_ = (*d*_*d*_ + *γ*4).

The basic reproduction number *ℛ*_*c*_ is difined as ${ℛ}_{c}=\bar{\varrho }\left(ℱ{𝒱}^{-1}\right)$, where ¯ denote the spectral radius of the next generation matrix $\bar{\varrho }\left(ℱ{𝒱}^{-1}\right)$. Thus,

(14) $$\begin{array}{c} R_{c}=R_{a}+R_{m}+R_{s}+R_{h}, \end{array}$$

where

(15) $$\begin{array}{c} R_{a}=\frac{\beta \left(1-\kappa \right)\left(1-\omega \right)f\theta_{a}}{K_{1}}\\ R_{m}=\frac{\beta \left(1-\kappa \right)\left(1-\omega \right)\left[\left(1-f\right)\left(\alpha +d_{\text{a}}+\gamma_{1}\right)+\xi \right]\theta_{\text{m}}}{K_{1}K_{2}},\\ R_{\text{s}}=\frac{\beta \left(1-\kappa \right)\left(1-\omega \right)v\left[\left(1-f\right)\left(\alpha +d_{\text{a}}+\gamma 1\right)+\xi \right]}{K_{1}K_{2}K_{3}},\\ R_{h}=\frac{\beta \left(1-\kappa \right)\left(1-\omega \right)\left[\eta_{1}K_{3}+\eta_{2}v\right]\left[\left(1-f\right)\left(\alpha +d-a+\gamma_{1}\right)+\xi \right]\theta_{\text{h}}}{K_{1}K_{2}K_{3}K_{4}}. \end{array}$$

The following result is obtained from Theorem 2 of [34] and is stated as:

**Theorem 2 .**

The infection-free equilibrium (DFE) of the model (2) is locally-asymptotically stable whenever *ℛ*_*c*_ < 1*, and unstable wheneverℛ*_*c*_ < 1.

The threshold quantity *ℛ*_*c*_ shown in equation (14) is the *control reproduction number*. *ℛ*_*c*_ measures the average number of new COVID-19 infections generated by a single infectious individual when introduced into a wholly susceptible population where basic public health mitigation strategies (such as social distancing, contact tracing, isolation, hospitalization etc.) are implemented [35]. It is apparent that the *control reproduction number* is a sum of the constituent reproduction numbers associated with four infectious classes (*I_a_,I_m_,I_s_,H*). That is, *ℛ*_*a*_ represent the reproduction number of new COVID-19 cases generated by infectious asymptomatic humans (with no symptoms), *ℛ*_*m*_ represent the number of new infections generated by symptomatically-infectious humans with mild symptoms, *ℛ*_*s*_ is the reproduction number of new COVID-19 cases generated by symptomatically-infectious humans with severe symptoms and *ℛ*_*h*_ is the reproduction number associated with the number of new COVID-19 cases generated by infectious hospitalized individuals. The epidemiological interpretation of Theorem 2 is that, there will be no COVID-19 outbreak in the population if the initial sizes of the infectious cases of COVID-19 are in the basin of attraction of the disease free equilibrium such that *ℛ*_*c*_ is less than unity.

## 4. Numerical Simulations and Results

In this section we shall conduct numerical simulation of the proposed COVID-19 model. It is imperative to note that when estimating model parameters, uncertainty may arise. Hence, we shall first conduct uncertainty and sensitivity analysis of the model parameters. Secondly, we shall carry out numerical simulation of our model with the aim of assessing two key objectives: (a) how various intervention strategies being implemented in Kenya to mitigate COVID-19 spread influence disease dynamics b) how will reinfection alter the long-term dynamics of COVID-19 in Kenya as well as globally. To achieve these objectives we shall numerically solve model equation (2) using MATLAB.

### 4.1. Uncertainty and Sensitivity Analysis

To identify the critical inputs of the COVID-19 epidemic model we perform sensitivity analysis so as to gain insights on how input uncertainties impact model outcome [36]. We conduct uncertainty and sensitivity analysis using the Latin hypercube sampling (LHS) technique which offers a comprehensive method for assessing model sensitivity to parameters over multidimensional parameter space [36]. One of the merits for using LHS technique in comparison to simple random sampling is that it requires fewer samples of parameters to achieve same accuracy (see [36] and the references therein for in-depth discussion on LHS). In our formulated COVID-19 model there are 25 parameter values. Thus, LHS technique becomes an important tool due to the relatively large uncertainty of the model parameter estimates used in conducting numerical simulation. The LHS technique works in synergy with the partial rank correlation coefficient (PRCC) which approximates the sign and strength of the relationship that exists between each model parameter and any specified output variable [37]. The PRCC values are only considered within a specific range, namely -1 and 1. The PRCC values that are near 1(-1) signal a strong positive (negative) correlation. Consequently, the relative importance of how model parameters influence model output is directly assessed by comparing the respective PRRC values [37]. The procedure for conducting uncertainty and sensitivity analysis requires two key steps. First, baseline parameter values are obtained. Then the lower and upper bound for each parameter in the model is set (see Table 3). After this multiple runs for a given outcome variable or response function is performed. For the proposed COVID-19 model the control reproduction number *ℛ*_*c*_ is selected as response function and to enhance accuracy 1000 random samples of parameter values are used.

**Figure 2:**
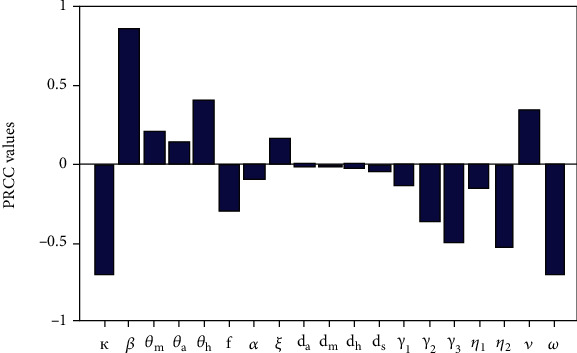
Represent the PRCC values for the control reproduction number *ℛ*_*c*_. The baseline parameter values used to generate the figure are depicted in Table 3.

Figure 2 depicts the sensitivity analysis of the control reproduction number *ℛ*_*c*_. It is clear that the effective contact rate *β,* the modification parameters (*θ_a_,θ_m_,θ_h_*), the rate at which infectious-asymptomatic humans progress to infectious-symptomatic class denoted by *ξ* and the rate at which infectious-symptomatic humans with mild symptoms progress to a class of individuals with severe symptoms denoted by *v* are all positively correlated to the control reproduction number. This implies that an increase in these parameters increases the *ℛ*_*c*_. Amongst these positively correlated parameters the contact rate *β* has the highest PRCC values suggesting that non-pharmaceutical intervention programs being implemented to mitigate COVID-19 spread should target on reducing social/physical contacts within the community. Hence, maintaining the recommended minimum social distance can have a positive impact. Furthermore the parameter *v* which account for the progression of humans with mild COVID-19 symptoms to a class of individuals with severe symptoms has a high PRCC value, suggesting that intervention measures such as hospitalization of these individuals can be implemented to reduce COVID-19 spread. The parameters that are negatively correlated to the control reproduction number *ℛ*_*c*_ include: *κ,ω,η*_1_*,η*_2_*,γ*_1_*,γ*_2_*,γ*_3_*,f,α,d_a_,d_m_,d_h_,d_s_.* Thus, increasing these parameters decreases the control reproduction number. The parameters *κ* and *ω* which, respectively, account for the proportion of humans who wear face masks and the proportion of individuals who observe the minimum physical/social distance are highly negatively correlated to *ℛ*_*c*_ Thus, the intervention measures such as face mask and observing social distance as being implemented worldwide by governments can significantly reduce the spread of COVID-19. Moreover, hospitalization of both individuals with mild and severe symptoms of COVID-19 can have a positive impact in controlling COVID-19 as suggested by their high negative PRCC values.

## 5. Model Fitting

**Figure 3:**
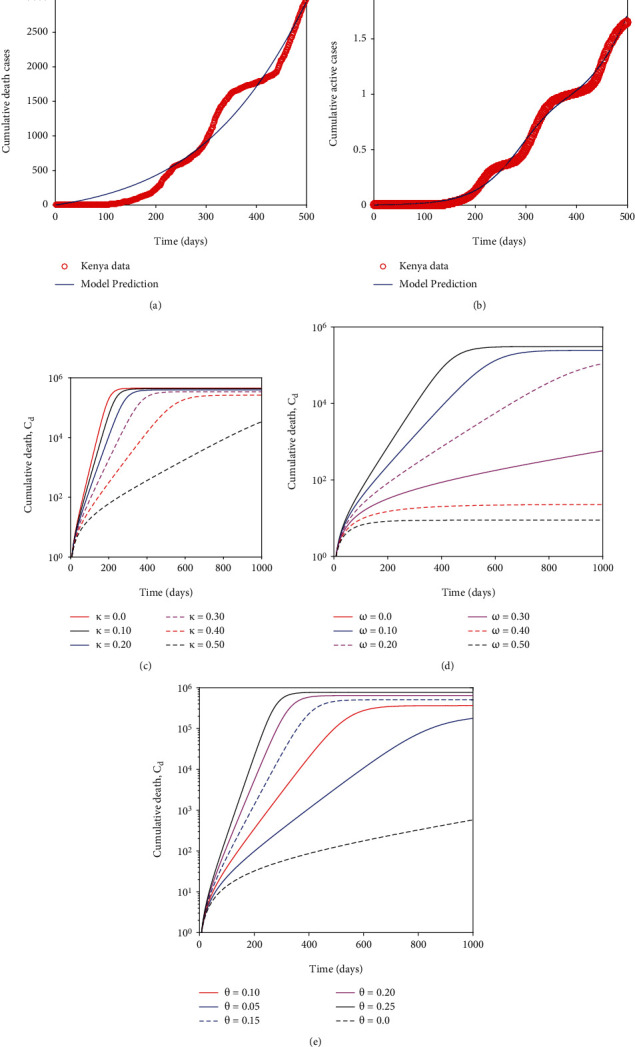
(a) Projections for the cumulative number of reported deaths. The baseline parameters used and the predicted parameter values are shown on Table 4. (b) Represents the projection of the cumulative reported active cases. (c) Illustrate maintaining social/physical distance will result in a decline in mortality cases. (d) Show a decline in death cases as the proportion of those wearing masks increases. (e) Depict the impact of reinfection on the long term dynamics of cumulative death cases. Figures (b), (c) and (d) are plotted using semi-logarithmic scale for clarity.

In this section we use the data for the daily reported death cases and new cases of infection to fit COVID-19 model inorder to estimate the model parameters. We chose initial conditions with the assumption that almost the entire Kenya population is susceptible to COVID-19. The initial data recorded suggest that there were three cases of COVID-19 as on March 3 2020, however we assume the disease was spreading undetected for sometime within the Kenyan population. Consequently, we use the following initial conditions; *S*(0)=47000000*,E*(0)=0*,I_a_*(0)=100*,I_m_*(0)=50*,I_s_*(0)=20*,I_d_*(0)=3*,H*(0)=0*,R_I_*(0)=0*,R_L_*(0)=0. The baseline parameters are obtained from the relevant COVID-19 literature. Figures 3(a) and 3(b) represent the projections of Kenya data fitted on model equation (2). It is clear that there is an exponential increase in the number of cumulative reported deaths and cumulative active cases. Note that cumulative death cases refer to the total number of deaths (caused by COVID-19) recorded within a specified period of time (interval). The total number of deaths increase on a daily basis given there are deaths recorded everyday. The cumulative active cases refer to the total number of COVID-19 cases recorded within a given time interval. The cumulative active cases increase on a daily basis since there are new infections recorded every day. The predicted model parameters shown on the Table 4 corresponds to a control reproduction number of about ≃ *ℛ*_*c*_ = 1.1117 which is greater than one, hence, suggesting that each infectious individual is able to transmit the COVID-19 to more than one person. The epidemiological implication of this is that in Kenya COVID-19 will continue to spread but at a slightly lower rate in comparison to other countries where *ℛ*_*c*_ is much higher (*>* 2 see [21]). The predicted model parameter values are then used to numerically investigate the key questions pertinent to this paper. That is the impact of reinfection on long term dynamics of COVID-19 in Kenya.

## 6. Impact of Non Pharmaceutical Interventions (Wearing Face Mask and Maintaining Physical/Social Distance) in Kenya

The Kenya data project that about 34.4% of the members of the public correctly and consistently wear masks in public and about 50% strictly observe and maintain social and physical distance (see Table 4). We now explore the epidemiological implication these non pharmaceutical interventions offer in mitigating COVID-19 proliferation in Kenya. It is evident from.

**Figure 4:**
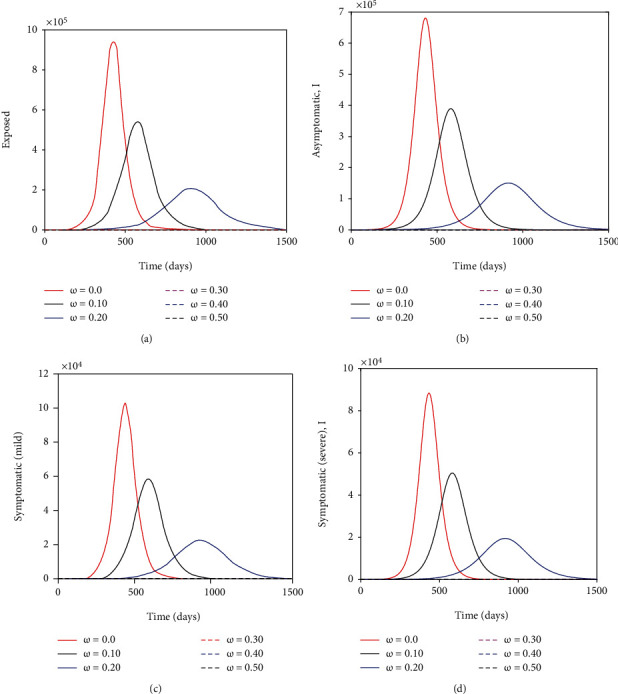
Show the impact of wearing face masks on the exposed, asymptomatic, symptomatic (with mild symptoms) and symptomatic (with severe symptoms) subpopulations. The parameter values used remain as shown on Table 4.

**Figure 5:**
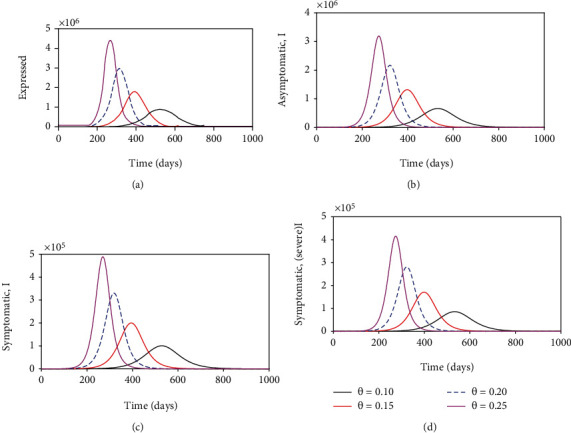
Show the impact of reinfection with COVID-19 on the exposed, asymptomatic, symptomatic (with mild symptoms) and symptomatic (with severe symptoms) subpopulations. The parameter values used remain fixed as shown on Table 4 except *θ* that is varied as shown in the figures.

Figure 4 that wearing face masks consistently and correctly significantly reduces the spread of COVID-19. With just over 30% wearing face masks the COVID-19 curve is much flatter than when 0% wear a face mask. Besides flattening the curve, an increasing percentage of those who wear face masks delay the peak of the epidemic outbreak in Kenya (see Figure 4). This implies that the health facilities will not be overburdened and will adequately manage the infectious cases. Moreover, it is imperative to note that when the entire general public does not adhere to wearing a face mask, the number of individuals with severe symptoms or critically ill can be over *>*80,000 as illustrated in Figure 5(d). This value is much higher than for individuals with mild symptoms. This can be explained by the fact that individuals with mild symptoms can transition to the cohort of individuals with severe symptoms once their immune system is compromised by the coronavirus.

**Figure 6:**
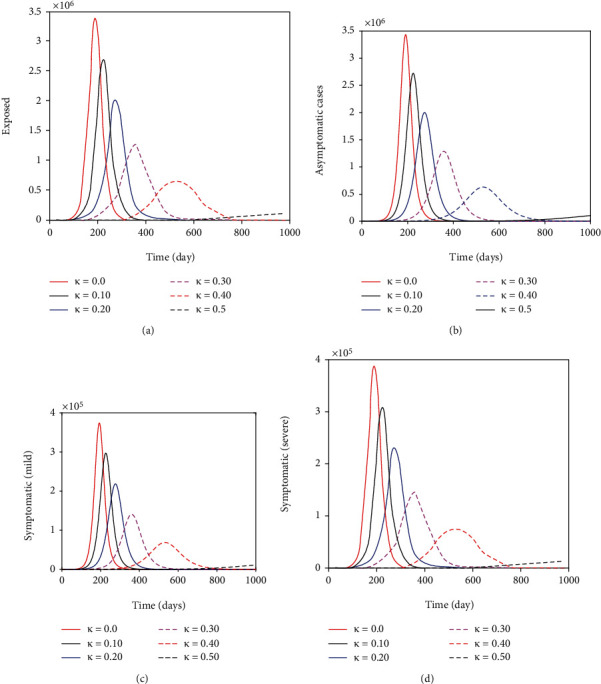
Show the benefit of maintaining physical/social distance on the exposed, asymptomatic, symptomatic (with mild symptoms) and symptomatic (with severe symptoms) subpopulations. The parameter values used remain as shown on Table 4 except *κ* that is varied as shown in the figures.

**Figure 7:**
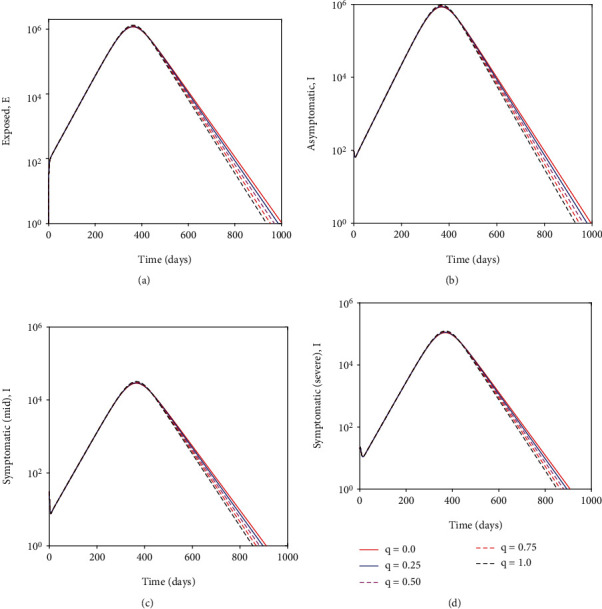
Depict the impact of quarantine/isolation on individuals suspected of being exposed to COVID-19, asymptomatic individuals, symptomatic individuals with mild symptoms and symptomatic individuals with severe symptoms. Semi-logarithmic scale is used along the *y*-axis for the purpose of clarity.

Figure 6 depicts that the non pharmaceutical intervention of maintaining physical/social distance within the general public has a positive impact. It can be observed that if Kenyans ignored Ministry of Health guidlines and maintained no physical/social distance the epidemic curve could have peaked after six months (on day 200-see Figure 7(d)). This could have resulted in overwhelmed health facilities and high mortality rate. In fact the number of individuals with severe COVID-19 symptoms could have reached *>*300000 (as suggested by Figure 7(d)) which is far much higher than Kenya health system capacity which has about 64,181 hospital beds across all sectors (i.e., private, public and faith based/NGO health facilities) [38]. This fast surge of COVID-19 could have been exacerbated further given that of the available hospital beds only about 58% (37,216) have oxygen supply fitted [38]. In general wearing face masks consistently and correctly and maintaining physical/social distance delayed, significantly the peak of COVID-19 in Kenya.  Figure 7 show the impact of quarantine and isolation of individuals suspected of being exposed with COVID-19. The Figure suggest that although quarantine is assumed to be an important control strategy of COVID-19, its impact in reducing COVID-19 is relatively low in comparison to wearing face masks (correctly and consistently) and maintaining social/physical distance.

Further, assuming there is no reinfection (i.e., *θ*=0) and varying equally the proportions of those who wear face masks and those who maintain physical/social distance, it is observed that wearing face masks correctly and consistently is more beneficial (as far as a decline in death cases are concerned) in comparison to maintaining physical/social distance. Although maintaining physical/social distance leads to a significant decline in death cases, the impact of *κ* in reducing death cases is slightly lower as compared to *ω.* This observation is suggested by Figures 3(c) and 3(d), respectively.

### 6.1. Impact of Reinfection and Detection of Asymptomatic Cases on COVID-19 Dynamics

During the early stages of COVID-19 outbreak there was scant information on its epidemiology. Some researchers suggested that recovered individuals acquired permanent immunity against reinfection. After a few months, empirical evidence established that this was not the case after some individuals who previously recovered, tested positive from COVID-19 virus. The research done by [14] which analysed antibody dynamics after infection shed some light on this debated issue by showing that about 50% of recovered individuals start losing their antibodies after a duration of six months.  Figure 3(e) suggest that if reinfection coefficient, *θ* increases, the mortality cases will profoundly increase to larger values than the one presently shown by Kenya data of about 3000 cases when the reinfection coefficient is predicted to be relatively small (≅ 0.05).  Figure 5 suggests that if high reinfection occurs (*θ*=0.30) within the community, the COVID-19 epidemic curve will peak on day 200. Further, on day 200, there will be about 4,500,000 individuals exposed to the COVID-19 virus, 3,000,000 asymptomatic cases, 490,000 individuals exhibiting mild symptoms and 400,000 individuals manifesting severe symptoms that require them to be hospitalized at the intensive care unit. Again this high number of critically ill patients outnumbers the hospital beds available within Kenya health system.

**Figure 8:**
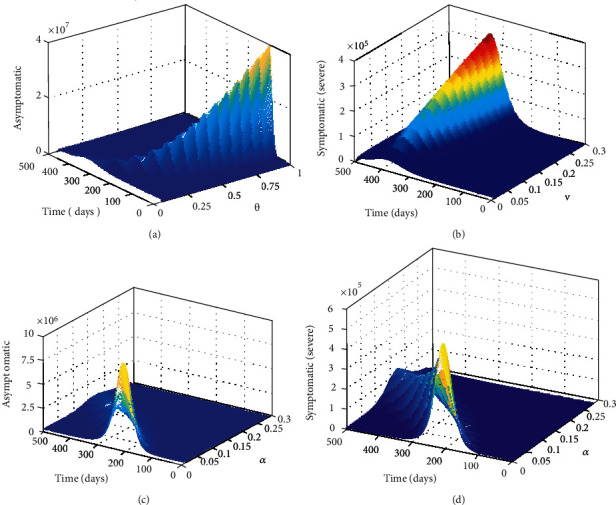
(a) Show the impact of increasing the reinfection coefficient on asymptomatic subpopulation. (b) Show the effect of increasing the rate of conversion of symptomatic individuals with mild symptoms to symptomatic individuals manifesting severe COVID-19 symptoms. (c) Show time series of asymptomatic individuals as a function of the detection rate (*α*). Increasing detection rate of asymptomatic cases and isolating them can significantly reduce the asymptomatic individuals who are silent spreaders of COVID-19. (d) Show time series of symptomatic individuals as a function of detection rate (*α*). There is an indirect relationship between removal of the asymptomatic cases from the general population and a reduction of symptomatic individuals who are critically ill and need urgent medical attention.

Moreover, if reinfection with COVID-19 occurs on a larger scale it will be catastrophic for many countries across the globe, both with weak and advanced health systems. This is because, if reinfection occur within the community, it will lead to a large pool of exposed individuals being exposed to the virus which will ultimately lead to a large pool of asymptomatic individuals, individuals with mild and severe symptoms, some of which require to be put on ventilator and supplementary oxygen. For instance, Figure 8(a) suggest that if reinfection coefficient *θ* approach one there will be approximately 40,000,000 Kenyans exposed to the virus.

Currently, it is not yet established how much protection previous infection or vaccination will offer. Mitigation measures such as (wearing face masks, maintaining social/physical distance and avoiding crowds) can still reduce the likelihood of being exposed to a second episode of COVID-19 (reinfection). Hence, adhering to non pharmaceutical interventions remains an important strategy in mitigating COVID-19 proliferation, especially in countries where vaccines are not affordable or available.  Figure 8(b) depict how an increase in parameter *v* will increase subpopulation of individuals with severe symptoms.

It is now apparent that reinfection will lead to a surge in mortality rate and accumulation of COVID-19 active cases which the Kenya health system can not handle. However, even in the presence of reinfection, the surge in COVID-19 cases can be prevented by interrupting the chain of progression of the virus through detecting asymptomatic individuals who silently transmit the disease. Given reinfection creates a large pool of asymptomatic individuals who as we know are capable of spreading the virus, mass testing of the general public to identify and isolate the asymptomatic cases can be effective in curbing COVID-19. As shown in Figure 8(c) increasing detection rate (*α*) through mass testing of the public can drastically reduce asymptomatic cases to almost zero. Reduction of asymptomatic cases as suggested in Figure 8(d) has an indirect relationship with individuals who are critically ill (or manifesting severe COVID-19 symptoms). This is because increasing detection rate of asymptomatic cases leads to a decrease of symptomatic individuals with mild symptoms and ultimately a reduction of individuals with severe symptoms. Currently, testing of the general public for COVID-19 is ongoing in Kenya but not on a large scale due to lack of adequate resources to conduct mass testing. As such those who are tested in Kenya health facilities consist of those individuals who present themselves to the health facilities with either mild or severe symptoms and also their closest contacts who are traced through contact tracing. However, this has been considered to have some loopholes as not all individuals who test positive for COVID-19 disclose all the people they have come into contact with. Consequently, community transmission of COVID19 continue to occur because individuals not detected through contact tracing remain in the general public. Furthermore, there has been shortages in supply of testing equipments and reagents, not only in Kenya but across Africa which largely depend on developed countries. Hence, large scale detection of asymptomatic individuals is again hampered.

## 7. Discussion and Conclusion

Reinfection with COVID-19 was a contentious issue during the early onset of COVID-19 pandemic. This followed from the fact that it was not understood whether initial infection offered an everlasting or partial protective immunity. As such some medical practitioners and researchers argued that previously infected individuals acquired “passport immunity” and therefore could be allowed to relax COVID-19 mitigation measures and mingle freely with the general populace [15]. However, further research involving serological testing for seasonal Human Coronavirus (HCoV-229E) found that the majority of patients lost 50% of the acquired antibodies after a duration of six months, 75% after a year and completely returned to baseline four years post-infection [14]. Based on this scientific information regarding reinfection with COVID-19 we developed a mathematical model with the aim of investigating how reinfection mechanisms will influence COVID-19 dynamics in Kenya. We categorized the infectious cohorts based on symptoms manifestation. Consequently, the proposed model has asymptomatic infectious individuals, symptomatic infectious individuals with mild symptoms and symptomatic infectious individuals with severe symptoms. First sensitivity and uncertainty analysis was conducted on the basic reproduction number and findings suggest that non pharmaceutical intervention measures such as wearing face masks and maintaining social/physical distance are effective in curbing the spread of COVID-19 as supported by a high negative PRCC values (-0.711 and -0.697, respectively). Other intervention measures such as hospitalization of both individuals manifesting mild and severe symptoms seem to be beneficial in reducing the basic reproduction number, with highest reduction occurring when individuals with severe symptoms are removed from the community and managed at hospital facilities. This is also confirmed by strong negative correlation of *η*_2_ on *ℛ*_*c*_.

Fitting the proposed model on the Kenya dataset, we obtained model parameter values which were used to numerically investigate the impact of variation of these parameter values on COVID-19 dynamics. The numerical findings indicate that if Kenya did not implement non pharmaceutical interventions the cumulative death cases could have surged to higher values than the current one of about 3000 cases. With just wearing of face masks and maintaining physical distance, COVID-19 peak infections have been significantly delayed. This has been strongly supported by the numerical simulations where parameters accounting for wearing face masks consistently and maintaining social/physical distance were varied while others remain fixed. In fact both the sensitivity and uncertainty analysis as well as time series simulations suggest that wearing face masks is more beneficial in comparison to maintaining social distance. However, combining both wearing face masks and maintaining social distance can be more effective in curbing COVID-19. However, in densely populated cities/countries (e.g., Mumbai in India) maintaining physical/social distance can be much of a challenge to a significant proportion of the populace in comparison to sparsely populated cities with underdeveloped infrastructure facilities. Thus, non pharmaceutical interventions have profoundly minimized the COVID-19 related demands on the health care system in Kenya.

Further, exploration on reinfection infection mechanisms suggest that an increase of reinfection with COVID-19 can lead to a surge of cumulative COVID-19 active cases. In particular there will be a large pool of asymptomatic individuals. Given asymptomatic individuals are also infectious, having such a cohort acting as silent spreader of COVID-19 can be detrimental to the general public as it is likely to result to a prolonged COVID-19 outbreak in Kenya or sporadic outbreaks. However, numerical simulations suggest that even in presence of reinfection, if Kenya increases the detection rate of those who are asymptomatic through contact tracing, mass testing and isolating the positive cases, COVID-19 surge can be averted. Detection of asymptomatic cases during the period when Kenya is not vaccinating the general public on a large scale can be effective in curbing COVID-19 proliferation. Furthermore, without adequate medical equipment and reagents being available in Kenya to test asymptomatic cases, it remains elusive to completely eradicate COVID-19.

## Figures and Tables

**Table 1:** Represent state variables of COVID-19 model equation (2).

*S*	Population of susceptible individuals
*E*	Population of individuals exposed to COVID-19
*I*_*a*_	Population of infectious asymptomatic individuals (manifest no symptoms)
*I*_*m*_	Population of infectious symptomatic individuals with mild symptoms (non-critical symptoms)
*I*_*s*_	Population of infectious symptomatic individuals with severe symptoms (critically ill)
*I*_*d*_	Population of infectious asymptomatic individuals isolated from the general population via contact tracing
*H*	Population of infectious individuals hospitalized due to COVID-19
*R*_*I*_	Population of recovered (tested negative) individuals with strong post COVID-19 protective immunity (protected against reinfection)
*R*_*L*_	Population of recovered individuals with weak or no COVID-19 protective immunity (partially protected against reinfection if exposed to COVID-19)

**Table 2:** Desription of model parameters.

Parameter	Description	Units
*β*	Effective contact rate (contact capable of leading to COVID-19 transmission)	Day^−1^
*ω*	The fraction of the members of the public who correctly and consistently wear masks in public.	Day^−1^
*κ*	Proportion of the population who strictly observe social/physical distance	Day^−1^
*θ*_*m*_, *θ*_*a*_, *θ*_*h*_	Modificationn parameters for the assumed reduction of infectiousness among infectious asymptomatic individuals, mild-symptomatic individuals and hospitalized individuals	Day^−1^
*q*	Measure of efficacy of contact tracing of asymptomatic humans and hospitalization in preventing COVID-19 transmission 0≤*q*≤1	Day^−1^
*f*	Proportion of exposed individuals who progress to infectious asymptomatic class after incubation period	Day^−1^
(1−*f*)	Proportion of exposed humans who progress to a class of infectious individuals with mild symptoms after incubation period	Day^−1^
$\frac{1}{\sigma }$	Intrinsic incubation period for individuals exposed to COVID-19	Day^−1^
$\frac{1}{\rho }$	Duration of COVID-19 protective immunity	Day^−1^
*ξ*	Rate at which infectious asymptomatic humans develop COVID-19 mild symptoms and move to *I_m_* class	Day^−1^
*α*	Represents the rate at which infectious asymptomatic individuals are detected via contact tracing, tested and isolated at home (home-based care) or quarantined such that they have no contact with the susceptible humans	Day^−1^
*v*	Rate at which infectious humans with mild symptoms develop severe symptoms and move to *is* class	Day^−1^
*γ*_*i*_{*i* = 1, 2, 3, 4}	Respectively, represent recovery rates for individuals in the *I_a_, I_m_, H, I_d_* classes	Day^−1^
*η*_1_ *,η*_2_	Respectively, represent the rate at which individuals with mild COVID-19 symptoms and individuals with severe COVID-19 symptoms are hospitalized	Day^−1^
*θ*	Modification parameter accounting for partial protection against COVID-19 reinfection among individuals with weak or no protective immunity 0*<θ*≤1	Day^−1^
*d_a_,d_m_,d_s_,d_d_,d_h_*	Respectively represent COVID-19 induced mortality rates for individuals in the *I_a_,I_m_,I_s_,I_d_,H* classes	Day^−1^

**Table 3:** Represents the baseline parameter values and the corresponding PRCC values.

Parameter	Baseline value	Range	*ℛ*_*c*_ PRCC values
*κ*	0.5	0.01−0.8	−0.697
*β*	0.5	0.05−1.5	+0.853
*θ_m_*	0.48	0.1 − 0.75	+0.193
*θ_a_*	0.48	0.1 − 0.75	+0.127
*θ_h_*	0.48	0.1 − 0.75	+0.425
*f*	0.8	0.1 − 0.95	−0.269
*α*	0.1168	0.05−0.25	−0.118
*ξ*	0.0025	0.05−0.15	+0.105
*d_a_*	0.0008	0.001−0.008	−0.010
*d_m_*	0.0008	0.001−0.008	−0.016
*d_h_*	0.0016	0.001−0.008	−0.026
*d_s_*	0.0016	0.001−0.008	−0.034
*γ*_1_	0.05	0.01−0.25	−0.115
*γ*_2_	0.05	0.01−0.25	−0.351
*γ*_3_	0.05	0.01−0.25	−0.517
*η*_1_	0.20	0.01−0.35	−0.166
*η*_2_	0.20	0.01−0.35	−0.526
*v*	0.15	0.01−0.25	+0.345
*ω*	0.3	0.01−0.8	−0.711

**Table 4:** Represent the baseline parameter values and the corresponding predicted parameter values using Kenya COVID-19 data.

Parameter symbol	Baseline value	Source	Data predicted value
*κ*	0.5	Estimated	0.511
*β*	0.5	Estimated	0.575
*θm*	0.48	[39]	0.65
*θa*	0.48	[39]	0.581
*θh*	0.48	[39]	0.566
*q*	0.6	[21]	0.558
*σ*	0.196	[7]	0.183
*f*	0.80	[2, 29, 30]	0.707
*α*	0.1168	[21]	0.119
*ξ*	0.0025	[21]	0.002
*d*_*a*_	0.0008	[33]	0.0009
*d*_*m*_	0.0008	[33]	0.000786
*d*_*c*_	0.0008	[33]	0.000698
*d*_*h*_	0.0016	[33]	0.000023
*d*_*s*_	0.0016	[33]	0.000151
*γ*_1_	0.05	[28, 29]	0.0564
*γ*_2_	0.05	[28, 29]	0.0465
*γ*_3_	0.05	[28, 29]	0.0521
*γ*_4_	0.05	[28, 29]	0.0663
*η*_1_	0.20	[33]	0.234
*η*_2_	0.20	[33]	0.234
*ρ*	1/180	[14]	0.0056
*υ*	0.15	[21]	0.207
*ω*	0.30	Estimated	0.344
*θ*	0.01	Estimated	0.0533

## Data Availability

We used the world Health Organization Covid-19 data which is freely available: https://covid19.who.int/table or (https://www.worldometers.info/coronavirus/country/kenya/).
