# Correction to “Mathematical Modelling of COVID-19 Transmission in Kenya: A Model with Reinfection Transmission Mechanism”

**DOI:** 10.1155/cmmm/9781759

**Published:** 2025-09-26

I. M. Wangari, S. Sewe, G. Kimathi, M. Wainaina, V. Kitetu, and W. Kaluki, “Mathematical Modelling of COVID-19 Transmission in Kenya: A Model with Reinfection Transmission Mechanism,” *Computational and Mathematical Methods in Medicine* 2021 (2021): 5384481, https://doi.org/10.1155/2021/5384481.

In the article titled “Mathematical Modelling of COVID-19 Transmission in Kenya: A Model with Reinfection Transmission Mechanism,” there was an error in Figure 5a. The *Y*-axis was incorrectly labeled as “Expressed”; the correct label is “Exposed.” The corrected version is shown below as Figure 1.

We apologize for this error.

## Figures and Tables

**Figure 1:**
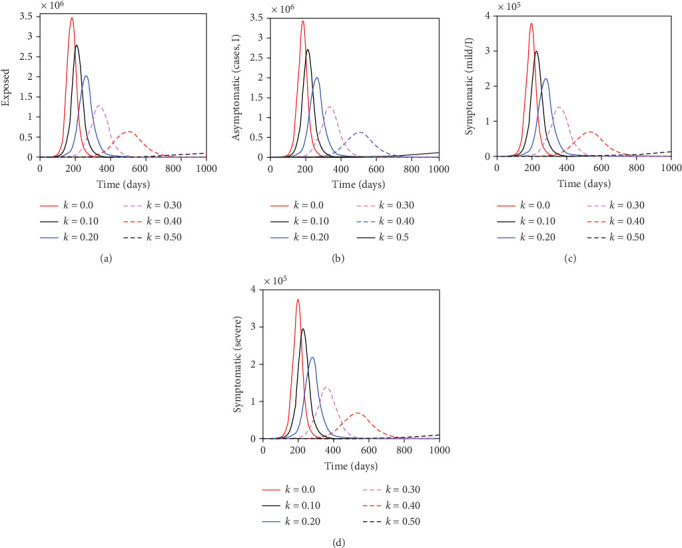
The impact of reinfection with COVID-19 on the exposed, asymptomatic, symptomatic (with mild symptoms), and symptomatic (with severe symptoms) subpopulations. The parameter values used remain fixed as shown on Table 4 except *θ* that is varied as shown in the figures.

